# Piezo-Resistive Properties of Bio-Based Sensor Yarn Made with Sisal Fibre

**DOI:** 10.3390/s21124083

**Published:** 2021-06-14

**Authors:** Ahmed Abed, Zineb Samouh, Cédric Cochrane, Francois Boussu, Omar Cherkaoui, Reddad El Moznine, Julien Vieillard

**Affiliations:** 1 GEMTEX–Laboratoire de Génie et Matériaux Textiles, University of Lille, ENSAIT, F-59000 Lille, France; zineb.samouh@ensait.fr (Z.S.); cedric.cochrane@ensait.fr (C.C.); francois.boussu@ensait.fr (F.B.); 2 Centrale Lille Institut, F-59000 Lille, France; 3 Laboratory REMTEX, ESITH, Route d’El Jadida, km 8, BP 7731 Oulfa, Casablanca, Morocco; cherkaoui@esith.ac.ma; 4 Laboratory Physics of Condensed Matter (LPMC), Faculty of Science El Jadida, Chouaib Doukkali University, El 24000 Jadida, Morocco; elmoznine@yahoo.fr; 5 COBRA (UMR 6014), UNIROUEN, INSA Rouen, Normandie Université, CNRS, 55 rue Saint Germain, F-27000 Evreux, France; julien.vieillard@univ-rouen.fr

**Keywords:** sisal yarn, piezo-resistive strain sensor, electromechanical properties, bio-based composite, in-situ monitoring

## Abstract

In this work, a sensor yarn based on a natural sisal yarn containing a non-electro-conductive core impregnated with PVA polymer and coated by PEDOT:PSS polymer as an electro-conductive sheath was investigated. The main objectives include the development of this new sensor yarn as a first step. Then, we look towards the insertion of this sensor yarn into different woven structures followed by the monitoring of the mechanical behaviour of composite materials made with these fibrous reinforcements. The combined effect of the structural geometry and the number of PEDOT:PSS coating layers on the properties of the sensor yarns was investigated. It was found that the number of PEDOT:PSS coating layers could strongly influence the electromechanical behaviours of the sensor yarns. Different methods of characterization were employed on strain-sensor yarns with two and four coating layers of PEDOT:PSS. The piezo-resistive strain-sensor properties of these selected coating layers were evaluated. Cyclic stretching-releasing tests were also performed to investigate the dynamic strain-sensing behavior. The obtained results indicated that gauge factor values can be extracted in three strain regions for two and four coating layers, respectively. Moreover, these strain-sensor yarns showed accurate and stable sensor responses under cyclic conditions. Furthers works are in progress to investigate the mechanism behind these first results of these sisal fibre-based sensors.

## 1. Introduction

There exists an actual and forthcoming demand of “green composites”, due to increasing environmental aims to replace oil-dependent resource components with natural fibres for the next generation of composite materials [1]. The natural fibrous reinforcement of composite materials is often based on fabric structures (2D and 3D) made with bio-based yarns [2]. Natural fibres have low density, biodegradable, and sustainable characteristics compared to synthetic fibres [3,4]. Thus, the use of natural fibres is widely becoming the subject of a large number of studies [5]. Among all of these studies, Corbin et al. [6] have developed a quasi-unidirectional woven fabric, based on hemp fibres impregnated with derivative sugar-based resin to produce a 100% bio-based composite material. The result of this research work has highlighted the relative higher performance of hemp-based reinforcements compared to commercial flax-based reinforcements in terms of rigidities.

In order to enhance the mechanical properties of natural fibre composites, it is important to check these properties at each scale, especially at the yarn scale, due to the weather-dependent growth of vegetal fibres [7,8,9]. Consequently, variations in weather conditions may lead to irregular fibre dimensions, which greatly affect the resulted linear density of the natural yarn and then can reduce or limit their use in composite materials. According to Mittal et al. [10], few studies have investigated the ageing effect of natural fibres, their fatigue behaviour, and their failure mode when they are used into structural composite material or during the textile technology processes. As suggested by Zeng et al. [11], it may be useful to measure the effect of mechanical stresses applied on these natural fibrous reinforcements during the textile manufacturing process in order to check how the resulted properties are affected. Thus, it may be possible to insert sensing devices inside the fibrous reinforcement that could behave similarly to the structural fibrous material and then help to monitor or detect locally the applied load while the textile material is submitted to external mechanical stresses. As a result, these fibrous reinforcements with their embedded sensing devices could be also impregnated to detect some damage initiation of composite materials and ensure their health monitoring during their lifetime duty.

Among all the existing sensing devices, strain gauges (or strain sensors) could be a solution for the evaluation of mechanical strain by electrical response [12]. However, the introduction of these strain gauges inside a fibrous reinforcement poses a challenge during textile manufacture and could potentially be responsible for failure propagation inside the composite material, due to the difference of mechanical behaviours between the sensor yarn and the fibrous reinforcement. Hence why we have chosen to use the same material for the sensor yarn and the structural yarns to be used inside the fibrous reinforcement of the composite material.

Textile-based strain sensors that are made with yarns or fabrics have many properties, such as deformability and flexibility compared to rigid semi-conducting electronic and thin-film-based sensors. Strain-sensor yarns have many advantages, such as: (1) an easier insertion into different textile structures by weaving or knitting technologies to provide sensing function into the required directions; (2) the capacity to provide local measurements of strain of the textile structure; and (3) the different locations of measurement in the textile reinforcement. Recent works have related that the manufacturing of strain-sensor yarns can be achieved by the fibre spinning method [13,14] or by the coating method [15,16]. Bilotti et al. developed an extrusion technique to produce strain-sensing multi-walled carbon nanotubes containing thermoplastic polyurethane fibres [17]. Although the spinning methods can produce sensor yarns with many properties, such as fatigue resistance and stretch-ability, these processes appear complicated and lead to higher cost in comparison with the coating techniques. Coating processes are used to functionalize textile substrates with different advantages, such as the homogeneity and continuity of the coated layers, scalability, and low cost production [18]. Trifigny et al. [19] studied an approach which consists in the integration of piezo-resistive sensor made with E-glass yarns into a 3D woven fabric. This sensor yarn, based on PVA and PEDOT:PSS conductive polymers, was able to detect dynamic constraints applied on different warp yarns by sensing locally the elongation. This approach has been very useful to identify the various parameters influencing on the mechanical properties of the 3D fibrous reinforcement during static and dynamic stresses.

The majority of these coated strain-sensor yarns are fabricated by using some conductive materials such as graphite, graphene, and PEDOT:PSS. However, few works concerning the coating of conductive polymers onto a natural multifilament substrate to fabricate yarn strain sensors have been carried out. More attention should be paid on the heterogeneous cylindrical geometry rod with some grooves, cavities, and voids on the entire surface of the natural yarn [20]. Thus, it is of interest to investigate the fabrication of strain gauges based on sisal natural fibres coated with a PEDOT:PSS conductive polymer and their use as sensors inside composite materials.

The sisal yarn used for this study is extracted from a plant (Agave Sisalana Perrine) that grows in Morocco. Sisal fibre contains some specific structural and geometrical characteristics, such as a high hydrophilic rate, a roughness surface, and low-angle microfibrils. The sisal fibre has also some interesting properties such as high toughness, low density, flexibility, and biodegradability [21,22]. These properties are required for fibre to be used as a core for conductive coating polymers [23]. This natural fibre showed better adhesion and physical and mechanical properties when used as a reinforcement with PLA thermoplastic resin for composite materials [24,25,26]. The increase of sisal fibre content improves the mechanical and dynamic properties of the composite material. However, the use of sisal natural fibre as core for strain sensors can be limited due to their irregular geometry, chemical composition, and mechanical properties [27], which are mainly dependent on climate conditions and species properties.

According to the literature review, it seems that the local and in-situ mechanical behaviour of natural fibrous reinforcements used in composite materials has not yet been fully investigated. Therefore, in the present work, the durability of sisal fibres, used as reinforcement for bio-based composites, is evaluated. The investigation has been done to find the optimized structure of the strain-sensor yarns with a PVA-coated sisal natural core yarn and a PEDOT:PSS electro-conductive sheath. The physical and electro-mechanical properties of the sensor yarn are also presented and discussed.

## 2. Materials and Methods

### 2.1. Material Selection

A sisal yarn, with an average diameter of 2.6 ± 0.35 mm and a linear density of 3300 Tex, was provided by the Sonajute company. This was later extracted from a Moroccan agave plant. The sisal yarn consists of a set of monofilaments, which exhibited a non-uniform network of overlapped fibres and little connected micro-pore structures with a roughness surface. The average diameter of the monofilament was approximately 200.56 ± 31.64 μm. This set of sisal monofilaments was twisted to 80 twists/metre in order to improve the elasticity, stretchability, and strength of the resulting yarn.

Poly (vinyl alcohol) (PVA, Mw 9000–10,000, 78–82% hydrolysed) was purchased from Sigma–Aldrich. Poly (3,4-ethylenedioxythiophene): Poly (styrene sulfonate), also named PEDOT:PSS, was supplied by the Heraeus company. PEDOT:PSS is expected to contribute to the electro-conductive sheath of the sensor yarns thanks to its prominent electrical conductivity, excellent mechanical strength, and thermal properties, even though this sample is well known to have more sensitivity to moisture hydration.

### 2.2. Preparation of PVA Solution

Polyvinyl alcohol (PVA) is a water-soluble aggregate polymer containing many alcohol functions. It has demonstrated several advantages such as good adhesion property, stability, and dispersing behaviour. In this study, 6 g of PVA (1.25 g/mL of density) particles were put into de-ionized water (100 mL) for a duration of 1 h at 90 °C and stirred with a magnetic stirrer until the complete dissolution of all the PVA particles.

### 2.3. Fabrication of Sensor Yarns

The sisal yarn was cleaned through successive sonication Soxhlet glassworks cleaning with petroleum ether as a solvent over 3 h. This cleaning process was very useful to remove all the impurities trapped inside the natural sisal yarn, which can strongly influence the interfacial adhesion properties. The pre-cleaned sisal yarn was then impregnated into a PVA solution for 30 s and dried at 90 °C for 15 min.

**Figure 1:**
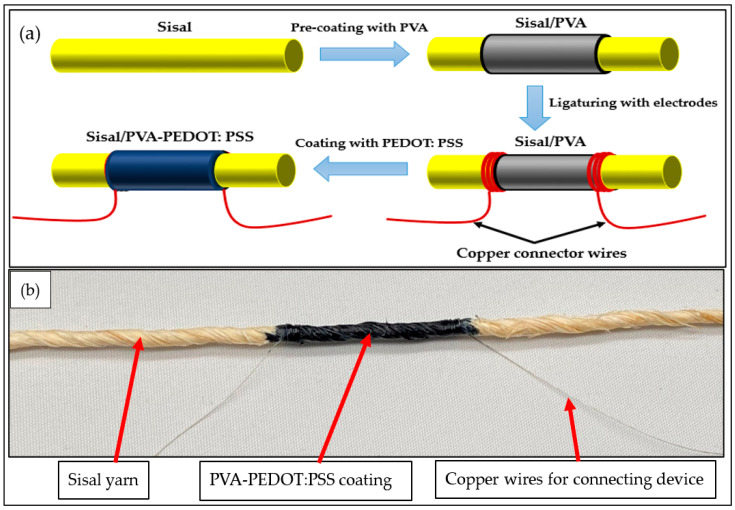
(**a**) Description of the fabrication process steps; (**b**) strain-sensor yarn.

The PVA content adopted for this experiment was elected at 6.0 wt%. Then, the resulting PVA-coated sisal yarn was legated by two copper wires used as electrodes. The diameter of the copper wires was 0.2 mm and the distance between these two ligatured copper wires was 30 mm. This distance value was chosen to be 10 times higher than the sisal yarn diameter to ensure more accurate measurement all along the yarn. To optimize the electrical contact between the PVA coated sisal yarn and the two ligatured copper wires, several PEDOT:PSS layers were coated and dried after each PEDOT:PSS layer deposit at 110 °C for 5 min. Different coating steps were carried out carefully to obtain the desired and optimized thickness of the electro-conductive PEDOT:PSS layers. It is important to state that the PEDOT:PSS layers were deposited under the same conditions. All steps involved in the fabrication of the sensor yarn are illustrated in Figure 1a,b.

In this study, we focused on two important factors such as the number of PEDOT:PSS coating layers as well as the geometry of the sensor yarns, in order to determine the optimal sheath thickness of the sensor yarn. The combined effects of these factors were investigated to explore the desired performance of sensor yarns, such as an accurate sensing performance and an optimal geometry.

Different number of PEDOT:PSS layers from 1 to 4 were coated onto the PVA-coated sisal yarn surface. For each layer of PEDOT:PSS, the mass and the thickness of the sensor were measured (Table 1). As can be seen from Table 1, the uptake amount of PEDOT:PSS tends to increase when the number of coated layers increases.

For this study, two layers and four layers of PEDOT:PSS coatings were selected for further investigation, which correspond to the compromise of medium and higher weight ratios of PEDOT:PSS, respectively.

### 2.4. Characterization of the Strain-Sensor Yarns

Sensor yarns were investigated via physical and chemical characterization in terms of morphology and structure analysis, mechanical strength, and electromechanical behaviours, as well as tensile, conductivity, sensitivity, repeatability, and dynamic stability.

The morphology and microstructures of the sensor yarns were further characterized by scanning electron microscopy (SEM). The SEM micrographs were obtained using a JEOL JEM-ARM200F HR setting. This analysis was performed on sensor yarns, before and after mechanical tests, to investigate the effects of the resulted deformation on sensor yarns.

The tensile properties of the sensor yarn were analysed using the MTS Criterion (2/M) testing system. The samples were tested according to the ISO 2062:2009 standard, with a coupon length of 200 mm, and submitted to an initial 10 N preload at 200 mm·min^−1^. These measurements were carried out at each step of the fabrication of the sensor yarn.

The electrical conductivity of the yarn sensors was measured on a Keithley 3705A digital multi-metre and calculated from the slope of I–U current–voltage curves. I–U characteristic responses were collected with contact points between the two copper wires of the sensor yarn and the two electrodes of the multi-metre device. The resistivity value was obtained in direct current (DC) mode with an applied voltage ranging between 0 V and +10 V with a step of 0.1 V. Then the conductivity of samples was calculated using Equation (1) [28]:

(1) $$\rho =\frac{R\times S}{l}$$

where R, S, and l are the resistance, the surface area, and the thickness of the sensor yarn, respectively. Both values of the surface S and the thickness l were measured on samples by using an optical microscope.

The electrical resistance changes (∆R/R0) of each sensor yarns were measured with a data acquisition system (National Instruments USB-6003) simultaneously on a tensile bench MTS Criterion. The real-time electrical resistance under a loading–unloading mechanical cycle was recorded online by a computer connected to a DAQExpress Processing Software synchronously and simultaneously. All the tests were performed at a strain rate speed equal to 200 mm·min^−1^ and different strain values up to strain at break. The electromechanical response of the sensor yarn is quantitatively represented by the gauge factor (GF). This later can be obtained from the linear slope between the relative electrical resistance (∆R/R_0_) and the mechanical strain (ε), according to Equation (2):

(2) $$\frac{\Delta R}{R_{0}}=GF\times \varepsilon$$

## 3. Results and Discussions

### 3.1. Morphological Properties of Sensor Yarn

**Figure 2:**
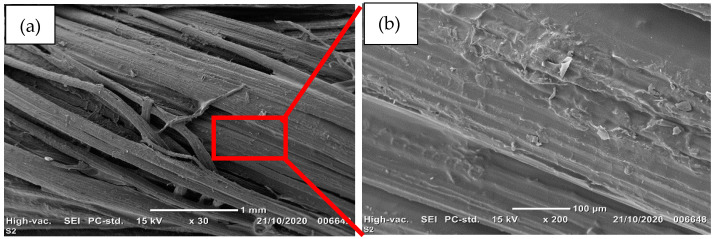
(**a**) SEM image of the surface of sisal yarn pre-coated with PVA. (**b**) Selected red lines area.

Figure 2 shows the SEM image of the surface of pre-coating PVA. After the pre-coating of the sisal fibres with the PVA layer, it can be observed that the cavities turned into a smooth and intact morphology. Moreover, the PVA-coated sisal yarn appears to be well impregnated, which could help with the deposit of the piezo-resistive sheet coating on its surface.

**Figure 3:**
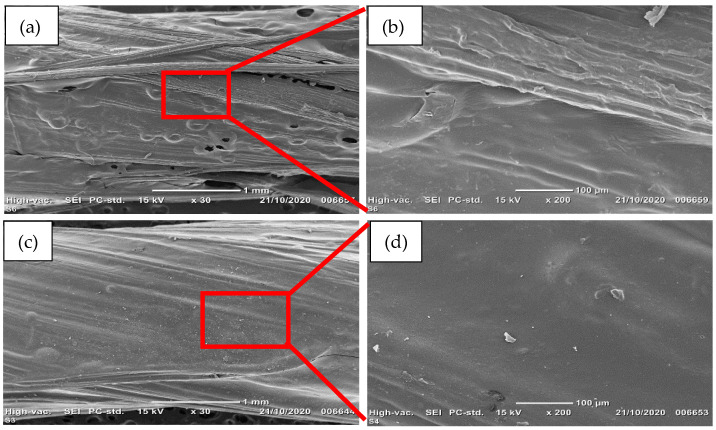
SEM images: (**a**) sensor yarn for two coated layers of PEDOT:PSS and (**b**) selected red line area. (**c**) Sensor yarn for four coated layers of PEDOT:PSS and (**d**) selected red line area.

The coating process of the PEDOT:PSS electro-conductive polymer aims at displaying a homogeneous and continuous film structure with a dense thickness layer about few microns around the PVA-coated sisal yarn. Figure 3 shows the SEM images of the four PEDOT:PSS-coated layers compared to the sensor yarn fabricated with two PEDOT:PSS-coated layers. These images suggest a successful deposition through the addition of PEDOT:PSS layers, which indicates that the polymer chains were successfully penetrated and fixed into the PVA crosslinking network to form a semi-interpenetrated network structure [29]. Thus, the PEDOT:PSS layers may also indicate that there is a good adhesion onto the surface of the PVA-coated sisal yarn. Furthermore, the gaps within the strain-sensor yarns are totally reduced, and the electro-conductive layer became thicker.

### 3.2. Electrical Characterization

**Figure 4:**
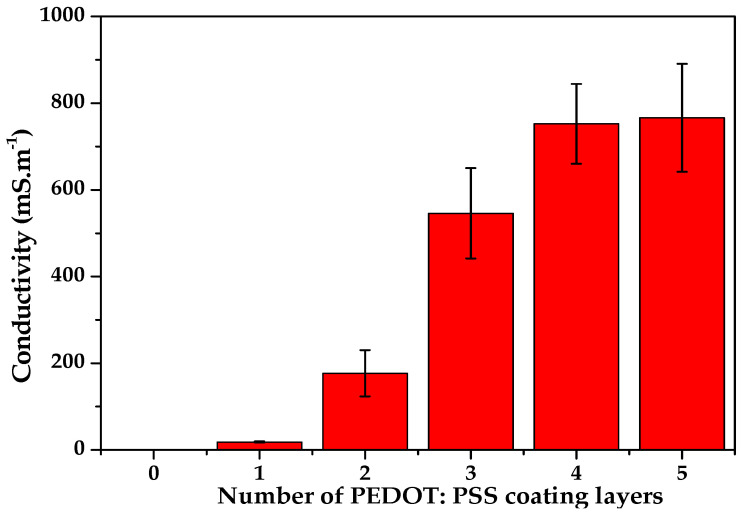
Electrical conductivity of the sensor yarn as a function of the PEDOT:PSS-coated layers.

Figure 4 shows the evolution of the electrical conductivity as a function of the number of PEDOT:PSS-coated layers from 0 to 5. It has been observed that the conductivity increased significantly when the number of PEDOT:PSS layers increased from two to four. The obtained conductivity value for 5 layers is very close to that for four layers. The value of the electrical conductivity increased from 176.71 mS.m-1 for the sensor yarn with two layers of PEDOT:PSS to 752.4 mS.m-1 for the sensor yarn with four layers of PEDOT:PSS.

When the number of coated layers of PEDOT:PSS is higher than two layers, the electrical conductivity increases sharply, which is referred to as the electrical pseudo percolation threshold network range of the sensor yarns [30]. Moreover, the electrical pseudo percolation threshold depends on the processing method, conductive micro-sheet type, and polymer matrix [31]. Generally, it was reported that an elastic core with an electro-conductive sheath near to the electrical pseudo percolation threshold showed good sensitivity due to the presence of conductive networks [12]. Therefore, these low electro-conductive layers could be sufficient to maintain the flexible properties of the sensor yarns and then enhance the strain-sensing performances for specific electro-mechanical applications.

Based on the above results, our study will be limited to the strain-sensor yarn with four coated layers of PEDOT:PSS in the following sections, due to the similar conductivity value obtained with 5 coating layers. This could be also an issue for reducing the amount of PEDOT:PSS used in manufacturing and consequently the material cost of the sensor yarns.

### 3.3. Mechanical Characterization

#### 3.3.1. Mechanical Properties of Sensor Yarns

**Figure 5:**
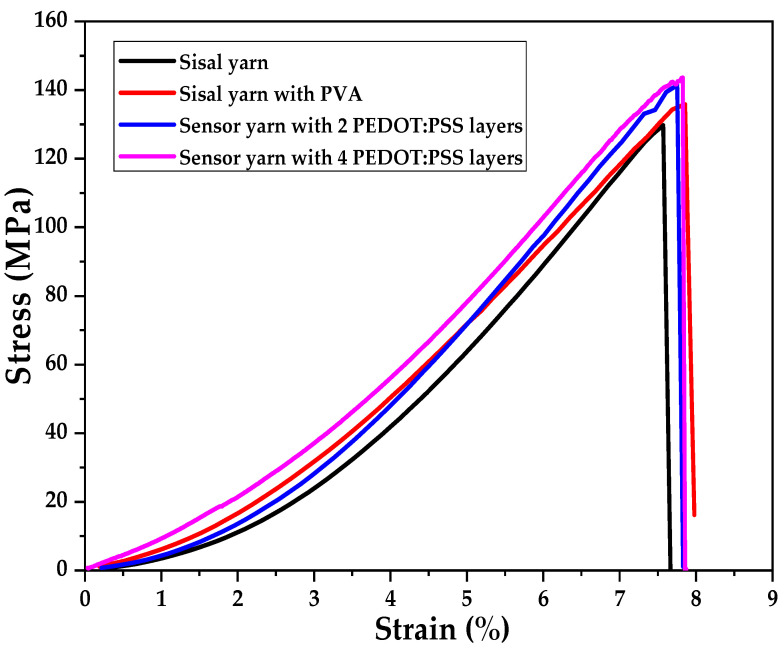
Stress–strain curves of sisal yarn alone, sisal yarn with PVA pre-coating, and sensor yarns with two and four coating layers of PEDOT:PSS.

The mechanical performance of the sensor yarn was investigated by applying a tensile load until rupture. Figure 5 shows the stress–strain curves of different yarns which help to analyse respectively the mechanical behaviours of a sisal yarn alone, a PVA-coated sisal yarn, and two sisal sensor yarns with two and four PEDOT:PSS-coated layers.

It can be seen that the strain-sensor yarn with highest number of PEDOT:PSS-coated layers (4) shows a slight increase of tensile strength. At the same time, the elongation at break increases with the increasing number of PEDOT:PSS-coated layers. Considering this result, it may be observed that the different number of coated layers applied on the sisal yarn did not affect the mechanical performance and behaviour of the initial material. The stress–strain curves reveal quite similar behaviour for a single applied load, and it could be interesting to check it according to cyclic loads.

The maximum values of tensile strengths were found 129.7 ± 5.6 MPa, 135.8 ± 0.6 MPa, 141.5 ± 0.6 MPa and 143.6 ± 1.3 MPa, respectively for the sisal yarns alone, PVA-pre-coated sisal yarn and sisal sensor yarns with two and four coated layers of PEDOT:PSS.

#### 3.3.2. Impact of the Applied Strain on Sensor Yarns

**Figure 6:**
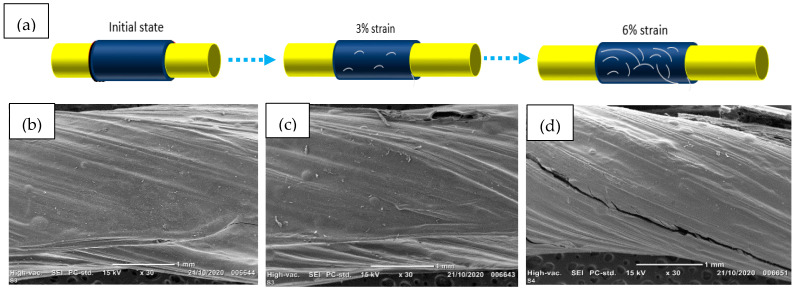
(**a**) morphological modifications of sensor yarns with four coated layers of PEDOT:PSS at different strain levels. SEM images of sensor yarns (**b**) without applied strain, (**c**) applied strain at 3% and (**d**) applied strain at 6%.

The effect of the applied strain on sensor yarns was investigated by SEM analysis. The morphological surfaces of the different sensor yarns corresponding to different values of elongation (0%, 3% and 6%) are represented in Figure 6. It can be observed that some micro-cracks were formed and propagated on the PEDOT:PSS sheath while the sensor yarns are longitudinally elongated. The micro-cracks have occurred, which could be due to lower value of PEDOT:PSS sheath elasticity than in the sisal yarn core. Figure 6c,d show some small micro-cracks when the sensor is extended to 3%, in comparison with 6% where further formation of larger micro-cracks can be observed. All these micro-cracks can strongly affect the change of the current conductive path of the PEDOT:PSS sheath and consequently its resistance values can also be modified [32]. Therefore, the variation of the micro-crack patterns from 3% to 6% of the strain of the sensor yarns could indicate a significant modification of their sensing performances.

#### 3.3.3. Mechanical Hysteresis

**Figure 7:**
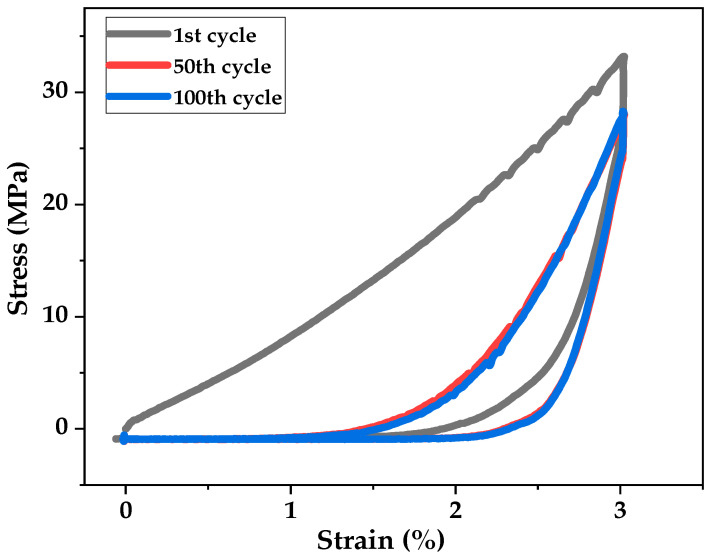
Mechanical hysteresis up to 3% strain for 1st, 50th and 100th cycles of sensor yarn with four coated layers of PEDOT:PSS.

The hysteresis properties of the sensor yarn with four coated layers of PEDOT:PSS was investigated by cyclic repetitive loading and unloading under the stepwise increase deformations of 3% at a slow rate of 200 mm·min^−1^ as reported in Figure 7. For the applied strain, it is observed a reduction of stress softness, mechanical hysteresis and Young’s modulus. Thus, it is noticed that most mechanical hysteresis and stress softening were occurred in the first cycle. In our strain-sensor yarn applications, the mechanical hysteresis and stress softening were generated from the irreversible elongations during loading issue to the slip between PEDOT:PSS microsheets and PVA-coated sisal yarn core chains. During the first stretching cycle, the inner bonds and instable contacts, formed during the process fabrication of the strain-sensor yarn, brake irreversibly during the relaxation. Therefore, the 50th cycle and all the following elongations need less power to reach the same deformations.

### 3.4. Electromechanical Properties of Sensor Yarn

#### 3.4.1. Uni-Axial Sensing Behaviour

The electromechanical properties of the strain-sensor yarns were also investigated. Mechanical cycles (stretching and releasing) were applied on strain-sensor yarn to measure the resulting change in the electrical resistance.

**Figure 8:**
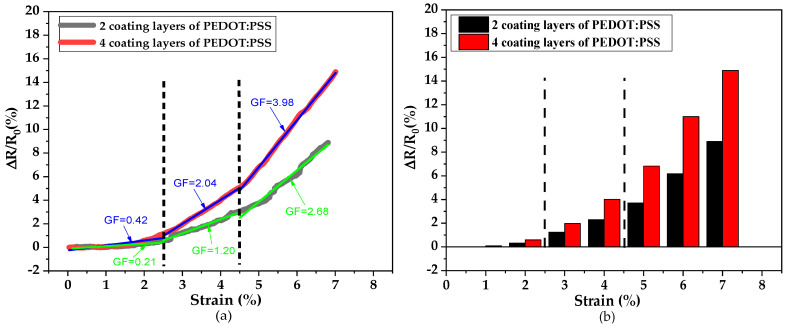
(**a**,**b**). Relative resistance variation with applied tensile strain for two and four coating layers.

Figure 8a,b shows the evolution of (∆R/R_0_) versus strain from 0 to 7% for sensor yarns with two and four PEDOT:PSS-coated layers. As it can be observed, the relative variations of resistance (ΔR/R_0_) of the sensor yarns with two and four coated layers have increased continuously with the applied strain, at a speed of 200 mm·min^−1^. However, the sensor yarn with two coated layers showed a failure at break for the maximum strain value of 7%. In contrast, the sensor yarn with four coated layers showed an elongation break up to 8% as shown in Figure 5.

The sensitivity of strain sensors is determined by the gauge factor (GF) which links the variation of resistivity to the elongation as represented in Equation (2) [33]. Thus, the (GF) gauge factor values of the different strain-sensor yarns have been calculated directly from stress–strain curves at different strain values.

In our case, the experimental data were fitted until the best linear fit was obtained according to Equation (2). A similar procedure was employed to extract the gauge factor (GF) values by Zheng et al. and Nakamura et al. [34,35].

The histogram presentation given in Figure 8b was very helpful in identifying the strain ranges with this linear behaviour. Figure 8a shows a better illustration for the choice of the more appropriate strain ranges. Three strain ranges were identified, and the obtained values of the gauge factor (GF) are given in Table 2.

It can be seen that the sensor yarn with four coated layers of PEDOT:PSS exhibited two different significant values of the gauge factor. The lower value, respectively 0.42 ± 0.02, corresponds to the first strain range between 0 and 2.5%, and the higher value, respectively 3.98 ± 0.01, corresponds to the second strain range between 4.5 to 7%. Then, the sensor sensitivity highly increases with the applied deformations. For the lower thickness value, especially for two coated layers of PEDOT:PSS, a lower value of electrical conductivity is expected, which can be explained by a lower gauge factor value at the same applied strain level. Regarding to the sensor yarn loadings, superior to the pseudo percolation threshold (i.e., four coating layers), the number of PEDOT:PSS layers are not enough to constitute a dense conductive network. When it has been elongated, the number of conductive paths is enhanced significantly compared to the conductive fibre with lower PEDOT:PSS loadings due to the reinforced contacts between the conductive network structures. This enhancement of network structures shows the highest sensitivity to stretch, reflected by an increase of gauge factor values at a high value of applied strain. Thus, the sensor yarns with four coated layers reveal a higher electrical conductivity and a high sensitivity to elongation.

#### 3.4.2. Cyclic Strain YARN Sensing Properties

**Figure 9:**
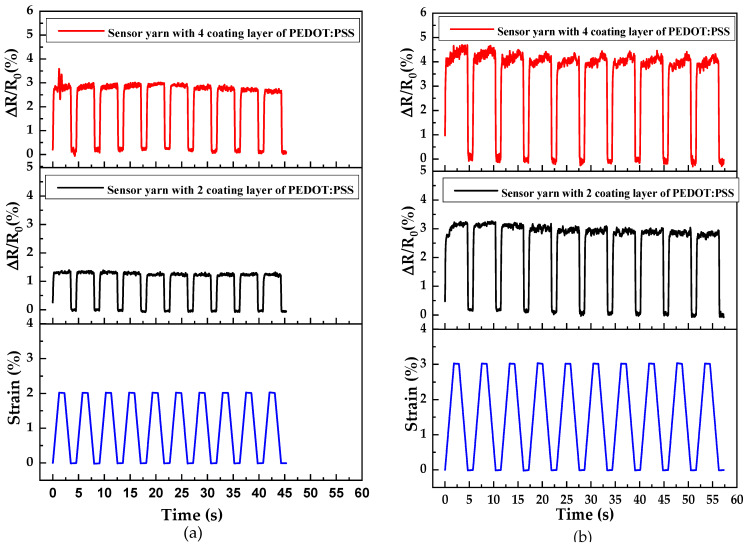
The relative variation of resistance (ΔR/R_0_) as a function of time under cyclic tensile load–unload for sensor yarns with different numbers of PEDOT:PSS-coated layers, for two ranges of strain between: (**a**) 0% and 2%; and (**b**) 0% and 3%.

The dynamic strain-sensing property is very important and desirable for wearable applications, thus the reliability of the bio-based sensor yarns should be also taken into account. The cyclic durability and reliability of these sensor yarns have been measured during a cyclic tensile loading–unloading process. Figure 9 displays the strain-dependence response for the sensor yarns with different PEDOT:PSS-coated layers under a cyclic stretching-releasing process. Figure 9a shows the relative variation of resistance (ΔR/R_0_) of the sensor yarns with two coated layers of PEDOT:PSS and where a strain of 2% for 10 stretching-releasing cycles was applied. At the first observation, the relative variation of resistance (ΔR/R_0_) and strain elongation pursue the same behaviour in each cycle. The sensor yarns are elongated with the increasing ΔR/R_0_ curve cycle. Thus, the first ΔR/R_0_ curve cycle shows a slightly higher value than the initial one after the first unloading, which suggests that the initial state of the electro-conductive PEDOT:PSS network structures could not be fully recovered due to electromechanical hysteresis. This phenomenon is caused by the mechanical hysteresis of the PVA-coated sisal yarn by the interaction between the PEDOT:PSS and PVA matrix. However, in the following cycles, it can be observed that the hysteresis became slightly inconsistent, which indicates that the electromechanical hysteresis effect only occurred in the first cycles, illustrating a good stability of the electro-conductive PEDOT:PSS networks of the sensor yarns.

Furthermore, it is notable that the peak values of the relative variation of resistance ΔR/R_0_ exhibit higher values for the sensor yarns with four coated layers of PEDOT:PSS under a strain of 2% (Figure 9a) compared to the sensing performances at 3% strain (Figure 9b). When the sensor yarns with four coated layers of PEDOT:PSS are extended to higher strain values of up to 3%, PEDOT:PSS particles experience large elongation with the deformation of the PVA-coated sisal yarn. A higher amount of these particles can lead to more serious destruction of the PEDOT:PSS electro-conductive networks. Meanwhile, the damage and construction of the electro-conductive network become very competitive and intense as more remarkable peaks of ΔR/R_0_ appeared.

**Figure 10:**
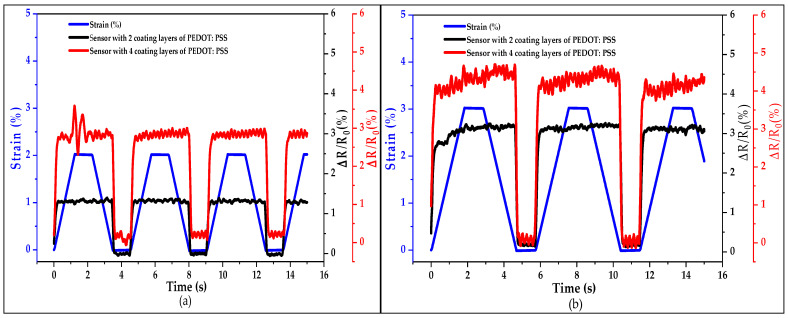
The ∆R/R_0_ versus time between 0 and 16 min under loading–unloading cycles for sensor yarns with two and four coated layers of PEDOT:PSS with strains of (**a**) 2% and (**b**) 3%.

To better investigate the distinction of the relative resistance ΔR/R_0_ with the loading–unloading strain shown in Figure 9, the ∆R/R_0_ of the two sensor yarns with two and four coated layers of PEDOT:PSS for the first three cycles with strains of 2% and 3% is presented in Figure 10a,b, respectively. The ΔR/R_0_ curves follow the strain behaviour during the loading–unloading process. However, a fluctuation of the ΔR/R_0_ during the stretching-releasing deformation was observed, corresponding to the ‘shoulder’ behaviour of every loading–unloading cycle (as shown in Figure 10a,b). For the ΔR/R_0_ curve cycles, the shoulder value at the maximum is higher than the shoulder value at the minimum. This phenomenon might be related to the effect of the dimensionality of the sensor yarn.

**Figure 11:**
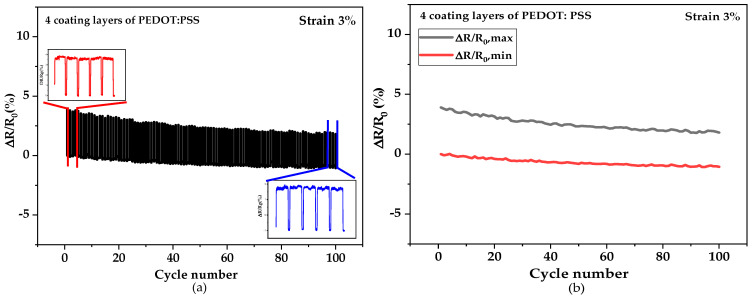
Multi-cycle elongation for the sensor yarns. (**a**) The ∆R/R_0_ of 100 stretching–releasing cycles with a maximum strain 3% at a slow rate of 200 mm·min^−1^ for sensor yarns with four coated layers of PEDOT:PSS; (**b**) The ∆R/R_0_ at strain 3% up to 100 cycles for strain-sensor yarns with four coated layers of PEDOT:PSS.

The ΔR/R_0_ signal outputs of 100 stretching–releasing cycles, performed at strain range values between 0–3% and at a strain speed of 200 mm·min^−1^, are shown in Figure 11a. A sharp drift down for the first initial cycles of the strain sensing response and then a stabilized trend can be observed, which is related to the constitution of an equilibrated state of the conductive networks after a period of adjustment [36]. Additionally, this could be due to the damage of the conductive networks and the hysteresis effect generated by the PVA viscoelastic matrix on the pre-coated sisal yarn. In the releasing mode, the variation of resistance ΔR/R_0_ exhibits slightly lower values than in the stretching mode, which seems to be similar to other flexible conductive sensing devices [37]. Figure 11b shows the variation of ∆R/R_0_ at the maximum and minimum strain of 3% up to 100 cycles for sensor yarns with four coated layers of PEDOT:PSS. Thus, the sensor yarns with four coated layers of PEDOT:PSS respond to cyclic stretching–releasing with efficient durability even after several initial cycles with a strain of 3%. The results seem to indicate a slight decreasing trend value and a stable response after numerous loading–unloading cycles for the sensor yarns with four coated layers of PEDOT:PSS.

## 4. Conclusions

In this study, a sensor yarn based on a PVA-coated sisal natural fibre utilizing PEDOT:PSS as electro-conductive sheath has been developed. These sensor yarns have been manufactured by using a coating technique, which has revealed several advantages, such as the simplicity to implement and the capacity to adjust, at low cost, the various electromechanical properties by adjusting the number of coated layers of PEDOT:PSS on the sisal yarn.

The sensitivity of the PEDOT:PSS-coated layers, for a given length value of 30 mm applied on the primary layer of pure PVA on the sisal yarn, has been improved thanks to the optimization of the manufacturing process. The combined effects of these two important material and fabrication parameters were investigated. The sensor yarns manufactured with four PEDOT:PSS-coated layers showed much higher electrical conductivity values.

This study revealed that the gauge factor (GF) of the sensor yarns increased with the increasing number of coated layers of PEDOT:PSS and reached an optimum value at four layers. In addition for the optimized four coated layers of PEDOT:PSS, the cyclic sensing behaviours of the sensor yarn were investigated for 100 cycles at a strain speed equal to 200 mm·min^−1^ and at an applied strain of 3%.

This sensor yarn has also showed high sensitivity with a gauge factor (GF) value equal to 3.98 ± 0.01 and stable responses, proving their efficiency for use in the fibrous reinforcement of composite materials for their health monitoring purpose.

Further studies are to be carried out to assess the use and insertion of the prepared bio-based sensor yarn with four coated layers of PEDOT:PSS into a natural reinforcement fibre for green composite application.

## Figures and Tables

**Table 1:** Mass and thickness of the PEDOT:PSS coating layers.

	PEDOT:PSS Coating Properties	
Number of Coating Layers	Mass (mg)	Thickness (µm)
1	18.7 ± 0.9	47.0 ± 1.6
2	22.5 ± 1.3	67.0 ± 2.3
3	26.5 ± 3.4	81.0 ± 3.0
4	29.7 ± 4.1	90.0 ± 3.3

**Table 2:** Electromechanical characteristics of sensor yarns: gauge factor (GF) value.

Samples: Sensor Yarn	Strain Range: 0–2.5%	Strain Range: 2.5–4.5%	Strain Range: 4.5–7%
	GF	GF	GF
**Two coating layers of PEDOT:PSS**	0.21 ± 0.01 (R^2^ = 0.791)	1.20 ± 0.01 (R^2^ = 0.982)	2.68 ± 0.01 (R^2^ = 0.985)
**Four coating layers of PEDOT:PSS**	0.42 ± 0.02 (R^2^ = 0.804)	2.04 ± 0.01 (R^2^ = 0.997)	3.98 ± 0.01 (R^2^ = 0.999)

## Data Availability

No data available online.
